# Connectomic Mapping of Chronic Musculoskeletal Pain: Neural Circuitries Identified Through a Systematic Review and ALE Meta‐Analysis

**DOI:** 10.1155/np/5301861

**Published:** 2026-05-08

**Authors:** Jeffeson Hildo Medeiros de Queiroz, Gabriel Mesquita da Conceição Bahia, Marcio Gonçalves Corrêa, Rebeca da Costa Gomes, Thais Alves Lobão, Erica Miranda Sanches Aires, Evander de Jesus Oliveira Batista, Gláucia Mota Bragança, Marta Chagas Monteiro, Carlomagno Pacheco Bahia

**Affiliations:** ^1^ Laboratory of Neuroplasticity, Health Sciences Institute, Federal University of Pará, Pará, Brazil, ufpa.br; ^2^ Laboratory of Protozoology, Tropical Medicine Nucleus, Federal University of Pará, Pará, Brazil, ufpa.br; ^3^ Laboratory of Oxidative Stress, Immunology and Microbiology, Health Science Institute, Federal University of Pará, Pará, Brazil, ufpa.br

**Keywords:** brain, chronic pain, functional magnetic resonance imaging (fMRI), musculoskeletal diseases, neuronal plasticity

## Abstract

Functional neuroimaging of the encephalon of humans with chronic musculoskeletal pain (CMP) has consistently demonstrated functional alterations in the neurophysiological properties of cortical and subcortical circuits. Nevertheless, the current knowledge on specific neural circuitries that may occur in different CMP subgroups is limited, which in turn limits the understanding of the encephalon mechanisms associated with persistent pain and clinical heterogeneity. This systematic review (CRD42022382309) and activation likelihood estimation (ALE) meta‐analysis of observational functional magnetic resonance imaging (fMRI) studies on human encephalon aimed to characterize specific patterns of connectomic reorganization in different CMP subgroups. PubMed, Web of Science, and Scopus databases were searched. Two independent reviewers read titles and abstracts, full texts, assessed methodological quality using the Newcastle–Ottawa scale, and extracted *X*, *Y*, and *Z* coordinates. The data analyses were conducted using the GingerALE 3.0.2 software and complemented by frequency analyses. All neuroanatomical coordinates used in the ALE meta‐analyses were standardized to the Montreal Neurological Institute 152 space. A 95% confidence interval (CI) for the family‐wise error (FWE) rate was applied using an initial uncorrected voxel‐level threshold of *p*  < 0.001, together with 1000 permutation tests and a minimum cluster volume of ≥200 mm^3^. In total, 43 studies out of 5543 records met the inclusion criteria and none presented a very high risk of bias. The seven encephalon regions comprising the basic neural circuitry of CMP (somatosensory cortex, motor cortex, anterior cingulate cortex, insula, prefrontal cortex, thalamus, and cerebellum) correspond to an integrated connectomic hub that coordinates sensory–motor discriminative, affective‐emotional, and cognitive‐motivational processing. Additionally, 13 other regions are specifically recruited in distinct conditions of CMP. These parallel connections expand the processing network and add dimensions related to reward, memory, descending pain modulation, and attention. This dynamic reorganization reflects a pattern of cross‐modal plasticity with maladaptive mechanisms.

## 1. Introduction

Chronic pain is an individual experience that integrates sensory, emotional, cognitive, and motivational components, and represents a significant global public health problem, with substantial clinical, social, and economic impacts [1–4]. Among the different existing chronic pain conditions, chronic musculoskeletal pain (CMP) is the most prevalent condition and the main cause of physical disability worldwide [2, 5]. The Global Burden of Disease (GBD) recently reported that the number of people with some type of CMP increased by 123% between 1990 (221 million) and 2020 (494 million) and may increase by 115% by 2050 (1.06 billion) [6]. Additionally, CMP is associated with a higher risk of suicide, dementia, and other chronic conditions, as well as reduced functional capacity and a greater chance of premature mortality [7]; therefore, a comprehensive understanding of the neurobiological mechanisms related to persistent CMP is urgently needed to improve care of affected individuals.

Scientific advances resulting from functional neuroimaging studies demonstrated complex encephalon alterations in humans with CMP, highlighting functional changes in the neurophysiological properties of cortical and subcortical circuits in areas classically related to the perception of pain experience, such as the insula, anterior cingulate cortex, prefrontal cortex, thalamus, and somatosensory cortex, areas consistently grouped within the concept of the “pain matrix” [8–14]. Changes of this nature may partially explain the persistence of pain and the decline in functional capacity of individuals with CMP, even in the absence of continuous noxious stimuli, indicating that CMP is a persistent condition resulting from the collective activity of various encephalon regions in conjunction with ascending and descending pathways, as well as supported by connectomic alterations [4, 15–17]. Despite these advances, current knowledge about the specific neural circuitry of CMP, as well as variations in this circuitry that potentially occur in different CMP subgroups, remains limited. This gap constrains the understanding of the encephalon mechanisms that underlie persistent pain, and its clinical heterogeneity observed among individuals, maintaining chronic pain perception as one of the enduring challenges in biology and medicine [4].

Functional magnetic resonance imaging (fMRI) has become an important tool for investigating encephalon alterations related to CMP through the identification of patterns of functional activity and connectivity, detecting the blood‐oxygen‐level‐dependent (BOLD) signal, functional connectivity (FC), the amplitude of low‐frequency fluctuation (ALFF), among other analysis [18–21]. The use of integrative approaches, such as activation likelihood estimation (ALE) meta‐analysis, provides the statistical combination of several fMRI findings and the mapping of consistent patterns of encephalon regions with significant spatial convergence of activation or connectivity [22–24]. Thus, the ALE meta‐analysis overcomes the limitations of individual studies, such as methodological heterogeneity and limited sample sizes of individual studies, providing a comprehensive connectomic mapping of encephalon regions and circuitries [22, 24].

This systematic review and ALE meta‐analysis of observational fMRI studies aimed to characterize specific patterns of connectomic reorganization in different CMP subgroups, testing the hypothesis that each CMP condition exhibits distinct patterns of connectomic reorganization. In this research, we not only delineated a neural circuitry common to all CMP conditions, but also identified differential patterns that potentially underlie the clinical and pathophysiological heterogeneity of the various CMP conditions.

## 2. Materials and Methods

### 2.1. Study Design and Protocol Registration

This is a systematic review of observational studies that used fMRI to assess functional changes in the human encephalon under different CMP conditions. The study protocol was registered in December 2022 in the International Prospective Register of Systematic Reviews database (CRD42022382309) and can be accessed at the following address: https://www.crd.york.ac.uk/PROSPERO/search. Minor adjustments were made to the protocol due to advances in the literature and theoretical deepening during the review process. Furthermore, this study was reported according to the updated recommendations of the Preferred Reporting Items for Systematic Reviews and Meta‐Analyses (PRISMA) [25, 26] and according to checklist for neuroimaging meta‐analyses [23]. These filled statements are available in Supporting Information 1 and 2.

### 2.2. Databases and Search Strategies

The research questions were based on the population/problem, exposure, control/comparison, and outcomes (PECO) framework [27]:

1. Which encephalon regions (O) constitute the neural circuitry of humans (P) with CMP (E)?
2. Which specific alterations are observed in this neural circuitry (O) in different CMP subgroups (E) in humans (P)?

Only studies including control groups were considered eligible. The comparison component (C) was operationalized through contrasts between control individuals and those with CMP, as reported across all selected studies.

Three independent researchers (Jeffeson Hildo Medeiros de Queiroz, Gláucia Mota Bragança, and Marcio Gonçalves Corrêa) searched observational studies published up to March 2025 in three databases (PubMed, Web of Science, and Scopus). The search strategy was developed using key concepts related to “brain” and “musculoskeletal pain,” incorporating both MeSH terms and free‐text terms. It was iteratively refined through pilot searches to optimize sensitivity and specificity. This procedure involved testing and adjusting search terms to ensure comprehensive retrieval across databases. Minor adaptations were made to tailor the search strategy to each database. Additionally, automated alerts were created to identify newly published studies. A detailed description of the search strategy is provided in the Supporting Information 3.

### 2.3. Study Selection

The records identified in the databases were imported into the Rayyan platform for duplicate removal and eligibility assessment [28]. Duplicate were removed prior screening. When the same study appeared more than once in the search results, only a single record was retained for screening and eligibility assessment.

The screening of studies was carried out in three stages: (1) reading titles and abstracts, (2) reading the full text, and (3) manually searching the reference lists of the selected articles to identify studies not found in the databases using our search strategy. The study selection process was independently performed by two reviewers (Jeffeson Hildo Medeiros de Queiroz and Gláucia Mota Bragança), who were blinded to each other’s decisions throughout all screening stages. Any disagreement between the researchers was resolved by arbitration by a third author (Carlomagno Pacheco Bahia).

### 2.4. Eligibility Criteria

Studies were selected for this systematic review only if they met all of the following inclusion criteria: (1) cohort studies or case–control studies (this criterion was applied to ensure the existence of a control group in the study to which data from individuals with CMP could be compared), (2) conducted in adult humans, (3) using fMRI, (4) whose experimental paradigm was performed with individuals at resting‐state or with sensorimotor stimuli, (5) published between 2002 and 2025, (6) in English, and (7) reporting functional or FC changes in the encephalon of humans with CMP. CMP was defined according to the International Classification of Diseases (ICD‐11) [3], encompassing primary and secondary CMP conditions, with persistent or recurrent pain for more than 3 months. In addition, eligible studies were required to (8) clearly specify the type of CMP for each exposed group and to exclude comorbidities that could influence brain function (e.g., severe psychiatric disorders or neurological diseases). No restriction was applied to the type of CMP; therefore, studies involving different CMP conditions were considered eligible.

Case reports, reviews, descriptive studies, opinion articles, technical reports, and clinical practice guidelines were excluded.

### 2.5. Risk of Bias in Individual Studies

Two co‐authors (Jeffeson Hildo Medeiros de Queiroz and Gláucia Mota Bragança) independently assessed the risk of bias of the selected studies using the Newcastle–Ottawa Scale [29], which is based on eight items distributed across three domains: (1) selection, (2) comparability, and (3) outcome for cohort or exposure for case–control studies [29]. Disagreements were resolved by a third co‐author (Carlomagno Pacheco Bahia). The studies were scored from 0 to 9 [29] and the risk of bias was categorized as very high (00–03), high (04–06), or low (07–09) [30]. The complete analysis of the risk of bias is available in the Supporting Information 4: Table S1, in the risk of bias assessment file.

### 2.6. Data Collection

The first author (Jeffeson Hildo Medeiros de Queiroz) obtained the data, and a second author (Carlomagno Pacheco Bahia) verified all extracted data. The following data were extracted from each selected study: (1) authors, (2) year of publication, (3) CMP condition studied, (4) study type (i.e., cohort or case–control), (5) sample size in each group, (6) risk of bias classification, (7) outcomes, (8) main results, and, when available in the selected studies, (9) the *X*, *Y*, and *Z* coordinates with which the meta‐analyses of this review were performed (Section 2.7). Also extracted was (10) the absolute frequency with which encephalon areas were activated in the different CMP conditions. All these data were categorized according to the CMP condition to enable subgroup‐specific analyses. The data collection spreadsheet is available in the Supporting Information 5: Table S2, in the data extraction file.

### 2.7. Data Analysis

For each CMP condition identified in this systematic review, both qualitative meta‐synthesis and ALE meta‐analysis were performed and independently described by two researchers (Jeffeson Hildo Medeiros de Queiroz and Carlomagno Pacheco Bahia). The meta‐synthesis was used to describe patterns of FC (e.g., increases or decreases), as reported in the original studies. It was derived exclusively from between‐group contrasts comparing control individuals and those with CMP (Supporting Information 6). In contrast, the ALE meta‐analysis was conducted to identify spatial convergence of reported peak coordinates.

When available in the selected studies, the coordinates of the neuroanatomical spaces were used to perform the ALE analysis for each CMP (Supporting Information 7). The activation coordinates from the original studies are available in the file “*X*, *Y*, and *Z* coordinates collected from primary studies” which can be found in the Supporting Information 7. ALE analysis quantifies the spatial convergence of activation foci or FC reported in different primary studies, treating them as three‐dimensional Gaussian probabilities centered on the *X*, *Y*, and *Z* coordinates [22, 31]. Therefore, this statistical method is useful for evaluating the activation probabilities for each voxel related to encephalon areas [22, 23]. We used the GingerALE 3.0.2 program from the BrainMap Database to perform ALE meta‐analyses, considering the random effects model and restricted to the standard gray matter mask [32]. The confidence interval (CI) used for the family‐wise error (FWE) rate was 95% (initial voxel‐level threshold *p*  < 0.001 uncorrected), the threshold permutations were 1000, and the minimum volume was ≥ 200 mm^3^ [22, 32–35].

All neuroanatomical coordinates used in the ALE meta‐analyses were standardized to the Montreal Neurological Institute 152 (MNI152) space. For studies that originally reported coordinates in Talairach space, we performed the conversion to MNI space using the Talairach Converter [35], which applies validated transformations that consistently approximate Talairach coordinates to MNI coordinates. In studies that provided MNI coordinates but without template specification, standardization was performed during the data preparation stage in GingerALE, selecting the option to align to MNI152 space, ensuring spatial compatibility among all included studies. Coordinates originally reported in MNI152 were used directly, without the need for additional transformations. This procedure ensured that all coordinates entered in the ALE analyses were represented in a single standard neuroanatomical space, increasing comparability between studies, reducing location biases, and ensuring the functional consistency of the probabilistic maps produced. Overlapping samples were not considered.

We used the Research Imaging Institute’s Mango version 4.1 program, and the anatomical brain model provided by GingerALE to visualize significant clusters, which guided us in preparing the meta‐analysis figures. The comprehensive Atlas of Cellular Resolution of the Adult Human Brain was used to identify the highlighted areas in the ALE meta‐analyses [36]. At last, to complement the findings of the ALE meta‐analysis, we determined the relative frequency of activation of encephalon areas. We obtained this result by dividing the number of times the area was shown to be functionally altered in the different CMP conditions identified in our review by the total number of CMP conditions identified, multiplying this value by 100, see Equation (1) [37]. Only areas that were activated in at least 7 of the 16 identified CMP conditions were included in the frequency analysis, which corresponds to the presence of functional alterations in that specific region in approximately 40% of the CMPs studied. A prevalence threshold of ≥40% (≥7/16 conditions) was applied to identify cross‐condition hubs in CMP. In datasets with multiple subgroups, low‐frequency occurrences often reflect stochastic or condition‐specific variability. Thresholds within the 30%–50% range are commonly used in ALE meta‐analyses to isolate stable network nodes. The ≥40% cutoff provides a conservative balance between sensitivity and specificity and operationalizes our distinction between core and accessory circuitry.

Identification of the relative frequency of functionally altered areas of the human encephalon under conditions of CMP.

(1) Relative frequency %= Number of times that the encephalon region was activated absolute frequencyTotal number of CMP conditionsn=16×100.

## 3. Results

### 3.1. Study Selection

**Figure 1:**
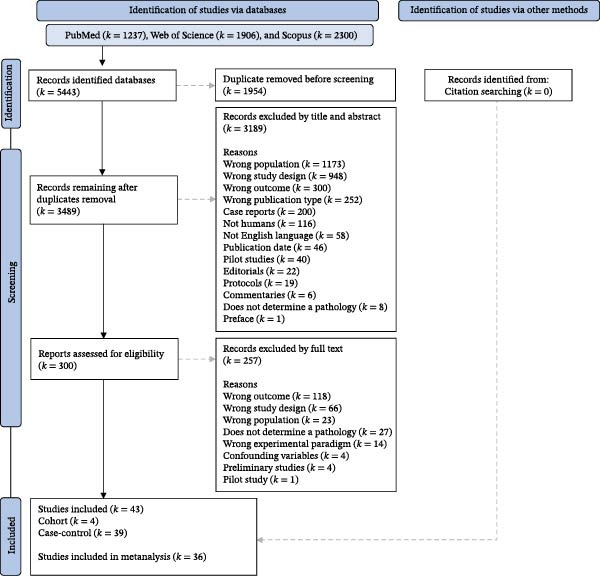
Flowchart of the systematic process for identifying, screening, determining eligibility, and selecting studies. *k*, Number of articles.

The flowchart presented in Figure 1 demonstrates the results of the search, screening, and study selection process. Initially, 5443 records were identified in the databases (Figure 1, identification). After removing 1954 duplicates, 3489 studies remained (Figure 1, identification and screening). In the title and abstract reading stage, 3189 studies were excluded (Figure 1, screening). Thus, 300 studies remained for full‐text reading (Figure 1, screening). At this stage, 257 studies were excluded (Figure 1, screening). Therefore, 43 observational studies were selected (Figure 1, included). Among these, four are cohort studies (*k* = 4) and thirty‐nine are case–control studies (*k* = 39), 9.30% and 90.69%, respectively (Figure 1, included). Of these, 36 studies met the necessary criteria for inclusion in the ALE meta‐analysis, representing 83.72% of the selected studies (Figure 1, included). The complete list of studies selected for this systematic review is available in the Supporting Information 8, in the list of studies selected file, and the complete list of abbreviations is available in Supporting Information 9.

### 3.2. Characteristics of Selected Studies

Functional alterations in the encephalon resulting from 16 CMP subgroups were identified and analyzed. Table 1 presents the distribution of the number of studies selected for each identified CMP condition. The sample sizes of the studies ranged from 12 to 165 participants, totaling 3067 participants included (1577 with CMP and 1490 controls). Chronic low back pain, fibromyalgia, and low back pain‐related leg pain were the most recurrent conditions among the selected studies (65.11%). The table for data extraction and study characteristics is available in Supporting Information 4.

**Table 1:** Distribution of the number of articles for each identified chronic musculoskeletal pain type (*k* = 43 studies).

Chronic musculoskeletal pain	Number of studies (*k*)
1. Chronic low back pain	14
2. Fibromyalgia	8
3. Low back pain‐related leg pain	6
4. Temporomandibular disorder	3
5. Chronic myofascial pain	2
6. Chronic cervical spondylotic pain	2
7. Adhesive capsulitis	2
8. Knee Osteoarthritis	1
9. Chronic ankle instability	1
10. Chronic whiplash‐associated disorders	1
11. Ankylosing spondylitis	1
12. Chronic idiopathic neck pain	1
13. Hand osteoarthritis	1
14. Massive irreparable rotator cuff tear	1
15. Rheumatoid arthritis	1
16. Anterior knee pain	1

*Note:* The total count exceeds the number of selected articles (43) since three studies [38–40] investigated more than one CMP subgroup. *k*, Number of articles.

### 3.3. Risk of Bias

**Figure 2:**
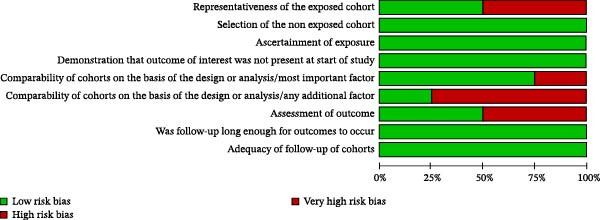
Risk of bias profile of the selected cohort studies (*k* = 4).

**Figure 3:**
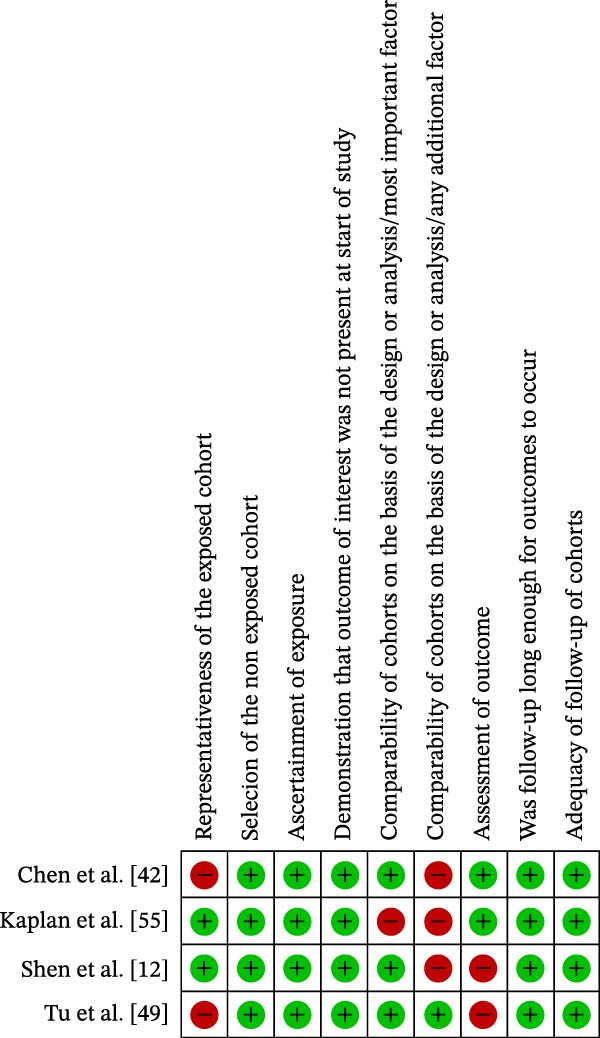
Risk of bias percentage for each item of the Newcastle–Ottawa Scale across cohort studies (*k* = 4).

**Figure 4:**
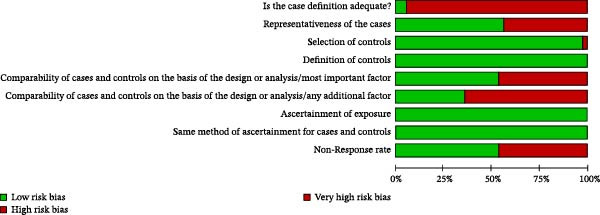
Risk of bias profile of the selected case–control studies (*k* = 39).

**Figure 5:**
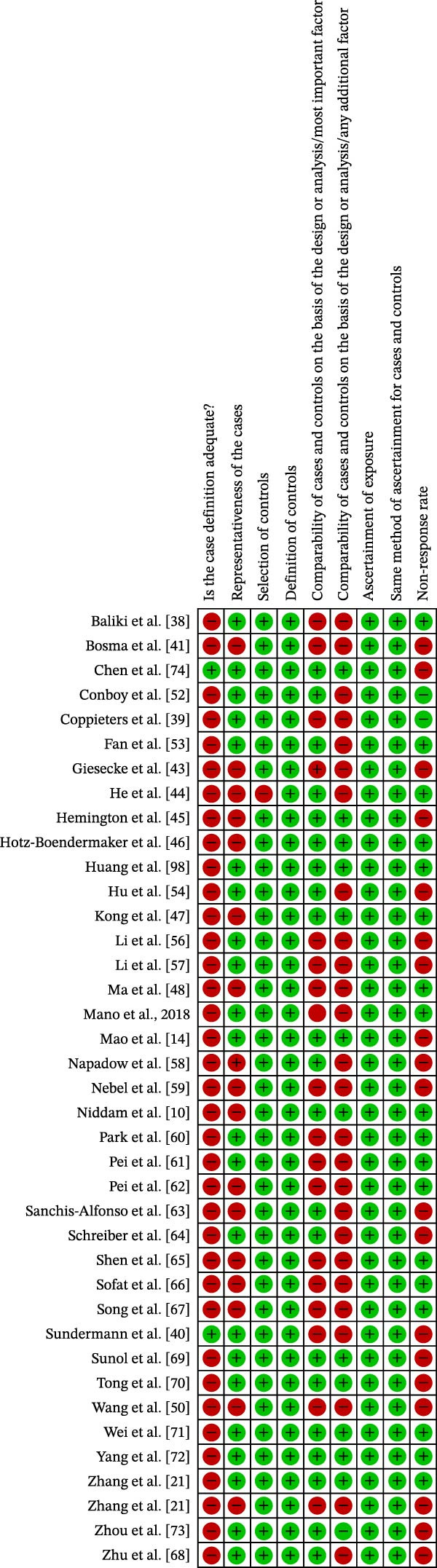
Risk of bias percentage for each item of the Newcastle–Ottawa Scale across case–control studies (*k* = 39).

The results of the risk of bias analysis are presented in Table 2, Figures 2–5, and in the Supporting Information 5. As demonstrated in Table 2, no selected study was identified in the very high risk of bias category. Nevertheless, some methodological limitations affected the overall quality of the selected studies, particularly in the following areas: (1) representativeness of cases, (2) comparability of cases and controls by important or additional factors, (3) assessment of outcome, and (4) case definition.

**Table 2:** Overall risk of bias classification among the selected studies (*k* = 43).

Low risk	High risk	Total
Cohort studies		
4 (100.00%)	0 (00.00%)	4
Case–control studies		
14 (35.89%)	25 (64.10%)	39

Regarding the “representativeness of cases,” some studies did not score because they did not use statistical estimates to determine the sample size [41–51] (Figures 2–5).

In the domain of “comparability of cases and controls by an important or additional factor,” many studies did not combine the analysis for important factors such as age or for additional factors such as sex, that is, male or female [12, 38–43, 48, 50–69] (Figures 2–5).

In the “outcome assessment” domain, some studies did not report the use of independent methods for assessing dependent variables [12, 49] (Figures 2–5).

At last, in “case definition,” most studies failed to achieve scores due to the absence of independent validation methods [10, 21, 38–41, 43–46, 50, 52, 53, 59–68, 70–74] (Figures 4 and 5).

### 3.4. Results of the Individual Studies: BOLD Signals and FC in CMP Cases

#### 3.4.1. The Neural Network in Chronic Low Back Pain

The fMRI findings demonstrated an increased BOLD signal intensity in the S1 and S2 cortices, inferior parietal lobe, and cerebellum [43], as well as an increased FC in the sensory, motor, and visual areas, rostral anterior cingulate cortices, posterior cingulate, orbitofrontal, angular gyrus, inferior frontal gyrus, middle frontal gyrus, insula, temporal lobe, lobule V, thalamus, and habenula [12, 14, 21]. Conversely, FC was decreased in the middle cingulate cortex, medial prefrontal cortex, lateral occipital cortex, precentral and medial frontal gyrus, paracentral lobule, salience network (SN; putamen, insula, anterior cingulate cortex, and caudate nucleus), habenula‐pons pathway, bilateral cerebellar crus II, and paracentral lobule [14, 38, 42, 49, 54].

#### 3.4.2. The Neural Network in Fibromyalgia

The BOLD signal intensities were increased in the anterior‐medial cingulate cortex, posterior parietal cortex, medial prefrontal cortex, supporting and presupporting motor cortices, ventral striatum, nucleus accumbens, frontal pole, cerebellum, and brainstem [41, 64]. Furthermore, the FC was increased in the dorsolateral prefrontal cortex, sensory and motor areas, thalamus, paracentral lobule, anterior insula, and bilateral cerebellum [68, 70, 71]. The fibromyalgia subgroup showed greater intrinsic FC within the default mode network (DMN) [58], while FC was decreased in the medial cingulate cortex, medial prefrontal cortex, nucleus accumbens, and basal ganglia [55, 60, 70]. Contrary to the earlier, Sundermann et al. [40] did not identify any significant difference between the FM group and controls.

#### 3.4.3. The Neural Network in Low Back Pain‐Related Leg Pain

The BOLD signal intensity was increased in the S1 cortex [61], and the FC was increased in the somatotopic subregions related to the lumbar spine, thorax, and face in the S1 cortex [61]. The FC was also increased in the middle frontal gyrus, superior frontal gyri, inferior parietal lobe, SN, and amygdala [50, 62]. In contrast, the FC was decreased in the ascending nociceptive pathway, DMN, and anterior cingulate cortex [50, 62, 72, 75].

#### 3.4.4. The Neural Network in Temporomandibular Disorder

The BOLD signal intensity was increased in S2, as well as the FC was increased in the posterior cingulate cortex, orbitofrontal cortex, parahippocampal gyrus, and striatofrontal regions [59, 76]. On the other hand, the FC was decreased in the dorsolateral prefrontal cortex, bilateral anterior cingulate cortex, amygdala, dorsal caudate nucleus, rostral dorsal putamen, insula, supramarginal gyrus, precentral gyrus, supramarginal gyrus, middle temporal gyrus, and thalamus [44].

#### 3.4.5. The Neural Network in Knee Osteoarthritis

The BOLD signal intensity was increased in the prefrontal cortex and precuneus, while the FC was decreased between the medial prefrontal cortex and the DMN [38].

#### 3.4.6. The Neural Network in Chronic Ankle Instability

The FC was increased in the S1, S2, M1, and M2 cortices, and decreased in the cingulate cortex, orbitofrontal cortex, precentral gyrus, middle frontal gyrus, middle temporal gyrus, and inferior parietal lobe [65].

#### 3.4.7. The Neural Network in Chronic Myofascial Pain

The BOLD signal intensity was increased in the S1 and S2 cortices, inferior parietal lobe, medial insula, and anterior insula [10].

#### 3.4.8. The Neural Network in Chronic Cervical Spondylotic Pain

The FC was decreased in the precuneus and medial prefrontal cortex [73].

#### 3.4.9. The Neural Network in Chronic Idiopathic Neck Pain and Chronic Whiplash‐Associated Disorders

The FC was increased in the amygdala and frontal operculum in women with chronic idiopathic neck pain and chronic whiplash‐associated disorders [39]; nevertheless, only patients with chronic whiplash‐associated disorders presented an increased FC in the orbitofrontal cortex, globus pallidus, and basal ganglia [39].

#### 3.4.10. The Neural Network in Adhesive Capsulitis

The FC was increased in the ventromedial prefrontal cortex, anterior cingulate cortex, S1 cortex, and thalamus [56, 57].

#### 3.4.11. The Neural Network in Ankylosing Spondylitis

The FC was increased in the posterior cingulate cortex, temporoparietal junction, DMN, and precuneus [45].

#### 3.4.12. The Neural Network in Hand Osteoarthritis

The FC was increased in the S1 and S2 cortices, thalamus, and cingulate gyrus [66].

#### 3.4.13. The Neural Network in a Massive Irreparable Rotator Cuff Tear

The FC was decreased in the motor area and visual cortex [52].

#### 3.4.14. The Neural Network in Rheumatoid Arthritis

No significant differences were observed between patients with or without rheumatoid arthritis [40].

#### 3.4.15. The Neural Network in Anterior Knee Pain

The FC was increased in the right supporting motor area and cerebellum I [63]. In anterior knee pain patients with catastrophizing thoughts (negative thought process that focuses on sensations of pain, [77]), an increased FC in the lateral parietal cortex, cerebellum II and VII, and globus pallidus was observed [63]. Conversely, the FC was decreased in the rostral prefrontal cortex, medial frontal gyrus, thalamus, and insula [63].

### 3.5. ALE Meta‐Analyses of Functional Alterations in the Encephalon of Different CMP Subgroups

**Figure 6:**
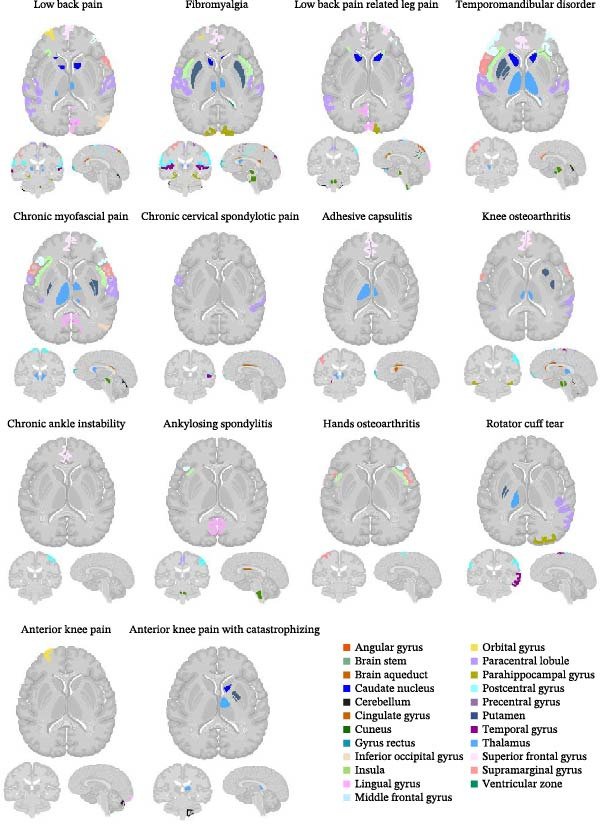
Statistical map from the ALE meta‐analysis demonstrating the significant convergence of the probability of activation of encephalon areas in humans under different conditions of chronic musculoskeletal pain (*k* = 36). The analysis demonstrated widespread functional alterations, with recurrent and consistent involvement of somatosensory areas, motor areas, anterior cingulate cortex, insula, prefrontal cortex, thalamus, and cerebellum (567 foci from 36 studies; MNI152 neuroanatomical space, cluster‐level FEW‐corrected, *p* < 0.05, and minimum cluster volume of ≥200 mm^3^).

The Figure 6 presents the results of the ALE meta‐analyses, which were based on peak coordinates derived from contrasts between control individuals and those with CMP. The results demonstrated that, despite the variations observed among CMP subgroups, a set of cortical and subcortical regions emerged recurrently and consistently, configuring a shared connectomic hub across conditions. This hub is composed of seven areas: (1) somatosensory cortex, (2) motor cortex, (3) anterior cingulate cortex, (4) insula, (5) prefrontal cortex, (6) thalamus, and (7) cerebellum. The table containing the X, Y, and Z coordinates from primary studies is available in Supporting Information 7.

### 3.6. Overall Frequencies of Functionally Altered Encephalon Regions in Different CMP Subgroups

The Table 3 presents the absolute and relative frequencies of the encephalon areas functionally altered in the different types of CMP. Note that the somatosensory cortex, motor cortex, and anterior cingulate cortex are the areas most frequently involved, followed by the insula, prefrontal cortex, thalamus, and cerebellum. Additionally, a set of other encephalon areas are specifically recruited in certain specific CMP subgroups, originating the additional accessory circuitries (Table 4).

**Table 3:** Regions of the human encephalon most frequently exhibiting functional alterations in chronic musculoskeletal pain (basic neural circuitry).

Encephalon regions	Absolute frequency	Relative frequency (%)
Somatosensory cortex	10	62.50
Motor cortex	8	50.00
Anterior cingulate cortex	8	50.00
Insula	7	43.75
Prefrontal cortex	7	43.75
Thalamus	7	43.75
Cerebellum	7	43.75
Total number of CMP subgroups studied = 16		

**Table 4:** Regions of the human encephalon specifically recruited in some chronic musculoskeletal pain subgroups (additional accessory neural circuitries).

Chronic musculoskeletal pain	Encephalon regions additional accessory identified
Chronic low back pain	Angular gyrus
	Calcarine córtex
	Habenula
	Intraparietal sulcus
	Medial frontal gyrus
Fibromyalgia	Entorhinal cortex
	Mesolimbic system
	Nucleus accumbens
Low back pain‐related leg pain	Ascending nociceptive pathway
	Brain stem
Chronic myofascial pain	Periaqueductal gray matter
Ankylosing spondylitis	Temporoparietal junction
Hands osteoarthritis	Cingulate gyrus
Anterior knee pain with catastrophizing thoughts	Middle frontal gyrus

*Note:* No additional accessory neural circuitry was observed in the other CMP subgroups not listed in this table.

## 4. Discussion

### 4.1. Main Discoveries

**Figure 7:**
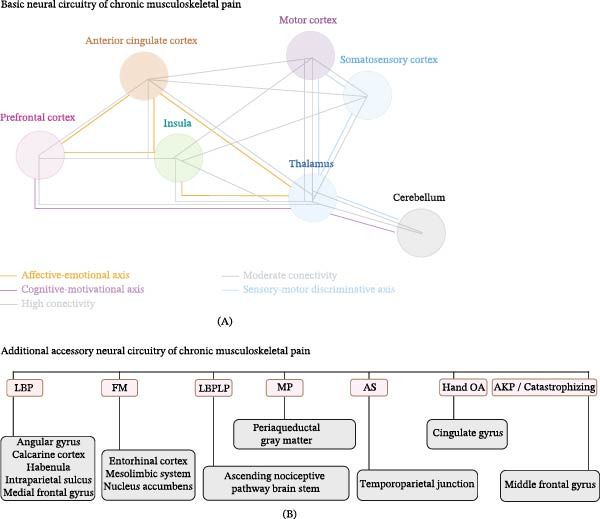
Basic and additional accessory neural circuitries of CMP: a connectomic model. (A) The thalamus acts as a central retransmission station that modulates the nociceptive flow within the basic neural circuitry, which is organized into three axes: the sensory–motor discriminative axis (blue lines) integrates the thalamus, somatosensory cortex, motor cortex, and cerebellum. The cognitive‐motivational axis (lilac lines) interconnects the prefrontal cortex, thalamus, and cerebellum. The affective–emotional axis (orange lines) interconnects the thalamus, anterior cingulate cortex, insula, and prefrontal cortex. (B) In specific subgroups of CMP, additional encephalon regions are incorporated into the basic neural circuitry, forming the additional accessory neural circuitries. In chronic low back pain (LBP), the angular gyrus, calcarine cortex, habenula, intraparietal sulcus, and medial frontal gyrus are incorporated into the basic neural circuitry. In fibromyalgia (FM), there is recruitment of the entorhinal cortex, the mesolimbic system, and the nucleus accumbens. In chronic low back pain related to the leg (LBPRLP), ascending nociceptive pathways are engaged in the basic neural circuitry. In myofascial pain (MP), the periaqueductal gray matter is incorporated into the basic neural circuitry. In ankylosing spondylitis (AS), the recruitment of the temporoparietal junction stands out. In hand osteoarthritis (Hand OA), the cingulate gyrus is added to the basic neural circuitry. In anterior knee pain (AKP) with catastrophizing, engagement of the middle frontal gyrus is observed (illustration created using BioRender/2025).

To the best of our knowledge, this is the first ALE systematic review and meta‐analysis to describe both basic and additional accessory neural circuitries of CMP. In total, seven encephalon areas comprise the basic neural circuitry, as they are consistently activated under CMP conditions, and another 13 areas are added to the basic neural circuitry in specific CMP subgroups, forming the additional accessory neural circuitries (Figure 7). A precise understanding of the encephalon areas responsible for processing the pain experience related to CMP and their corresponding neural circuitries, demonstrates that this neural processing is not restricted to somatosensory areas, but is also supported by activity and functional changes in multiple cortical and subcortical areas. This may contribute to the development of future strategies for CMP treatments that are more congruent with the processes occurring in the encephalon.

The basic neural circuitry of the CMP consists of the somatosensory cortex, motor cortex, anterior cingulate cortex, thalamus, insula, prefrontal cortex, and cerebellum (Table 3 and Figure 7A). Furthermore, some encephalon regions are specifically recruited and activated in specific CMP subgroups, constituting the additional accessory neural circuitries (Table 4 and Figure 7B).

### 4.2. Basic and Additional Accessory Neural Circuitries of CMP

The encephalon regions comprising the basic neural circuitry establish a neural network that integrates the sensory, motor, emotional, motivational, and cognitive components of CMP (Figure 7A). This corresponds to a common connectomic hub of involved areas, regardless of the etiology of CMP. The cross‐connectivity within the basic neural circuitry may be a key factor for pain persistence and reduction of physical capacity of individuals with CMP since these connections are correlated with pain intensity and reinforce its potential as a clinical biomarker [78]. Furthermore, robust neural connectivity suggests that these encephalon regions are synchronized to maintain a painful sensation even in the absence of nociceptive stimuli [78].

Among the encephalon structures that make up the basic neural circuitry of the CMP, the thalamus acts as a retransmission station for afferent nociceptive signals to the other components of the basic neural circuitry [79, 80] (Figure 7A). Moreover, it can act as a “modulator of nociceptive signal flow” within the CMP basic neural circuitry to increase or inhibit flow towards other connectome regions [80].

The somatosensory cortex plays a role in somatotopic pain discrimination, which is redirected to the motor cortex for planning and executing motor responses, as well as postural adaptations and protective movements, that is, movements aimed at relieving or preventing pain [8]. The interaction between the somatosensory cortex and motor cortex occurs through direct [81], indirect cortical pathways through the posterior parietal cortex [82], and subcortical pathways through the thalamus [82]. The cerebellum has a moderate FC with the thalamus and prefrontal cortex, which comprises the cognitive‐motivational axis of the CMP and suggests the integration between cognitive and motor aspects, as well as the mediation of attention and pain‐related coping strategies [15, 83] (Figure 7A). Additionally, the FC within the thalamus, somatosensory cortex, motor cortex, and cerebellum comprises the sensory–motor discriminative axis of CMP (Figure 7A).

The insula is essential for integrating sensory information with emotional and cognitive states and plays a key role in the subjective characteristics of the chronic pain experience [84–86]. Likewise, the anterior cingulate cortex is involved in the affective‐motivational processing of pain and mediates pain expectation, avoidance behaviors, and catastrophizing thoughts [85, 87]. The FC within the anterior cingulate cortex, insula, thalamus, and prefrontal cortex establishes the affective‐emotional axis for processing of chronic pain [84, 85, 87] (Figure 7A).

The remarkable overlap of pain matrix regions and the basic neural circuitry of CMP suggests a concomitant preservation of pain regions and long‐lasting functional reconfiguration of the pain matrix adapted to context of chronicity. Both networks (i.e., pain matrix and basic neural circuitry of the CMP) share pain processing regions, such as the somatosensory cortex, insula, anterior cingulate cortex, and thalamus [8, 9, 11]. Nevertheless, the pain matrix was originally determined by fMRI studies using acute pain and transient nociceptive stimuli to evaluate healthy participants, which only represents a temporary activation of regions associated with immediate pain responses [8, 9, 11]. Conversely, the basic neural circuitry determined in this study demonstrates patterns of neural activity consistently identified in humans with different CMP subgroups and suggests a functional reorganization based on the pain matrix. These findings suggest that the basic neural circuitry of CMP is a functional adaptation of the pain matrix supported by connectivity alterations inherent to the chronic state.

Some CMP subgroups specifically recruit other encephalon regions forming additional accessory neural circuitries (Figure 7B). These additional accessory areas integrate with the basic neural circuits, reflecting different neuroadaptive processes in specific subgroups of the CMP (Figure 7B). This dynamic reorganization of CMP circuitry reflects a conceptual pattern of cross‐modal plasticity, which refers to the nervous system’s ability to reorganize its FC in such a way that an encephalon region originally dedicated to a specific information modality is “recruited” or “reassigned” to process other information modalities or perform other functions [88]. These neural plastic alterations may represent maladaptive mechanisms that contribute to pain persistence or physical disability [89].

In chronic low back pain, the angular gyrus, calcarine cortex, habenula, intraparietal sulcus, and medial frontal gyrus are added to the basic neural circuitry (Figure 7B). These connectivity alterations in the DMN and visual/attentional networks suggest a greater coupling between body monitoring and visuospatial attention that may relate to hypervigilance of nociceptive signals in the lumbar region. Shen et al. [12] also suggested that the reorganization of the DMN and the visual network is a potential biomarker of chronic low back pain. The habenula is a core of the reward system and participates in the affective modulation of pain, in which aversive and appetitive components are amplified [90]. The middle frontal gyrus interconnects with the insula, anterior cingulate cortex, and thalamus, which may mediate the formation of additional accessory neural circuitry in individuals with chronic low back pain [91] (Figure 7B).

The following regions are recruited in fibromyalgia: entorhinal cortex, mesolimbic system, and nucleus accumbens (Figure 7B). The mesolimbic and nucleus accumbens alterations suggest a relationship between affective components and the reward system [92, 93]. Furthermore, the nucleus accumbens integrates the descending pain modulation system and influences the activity of the PAG and the rostral ventromedial spinal cord, which may explain the lower responsiveness to descending inhibition [92]. The entorhinal cortex supports the processing of contextual memory in persistent pain, which may favor the formation of negative pain‐related expectations [94] (Figure 7B). The regions comprising the additional accessory neural circuitry in low back pain‐related leg pain are the ascending nociceptive pathway and the brainstem (Figure 7B). Considering a radiculopathy condition, the prominent and increased activation of the ascending nociceptive pathway in the brainstem intensifies the projections to the basic neural circuitry [95]. The PAG, which is considered the main structure of the descending pain modulation system [96], is recruited in patients with chronic myofascial pain (Figure 7B). Alterations in the PAG suggest reduced responsiveness to descending modulation [96]. In ankylosing spondylitis, the temporoparietal junction constitutes the additional accessory neural circuitry. The temporoparietal junction is related to empathy and body perception related to pain [97] (Figure 7B). The recruitment of the cingulate gyrus in hand osteoarthritis explains the alterations in emotional/attentional regulation and behavioral response [87]. Specifically in anterior knee pain followed by catastrophizing, the middle frontal gyrus is recruited, highlighting that this area potentially mediates catastrophizing related to the pain experience in this condition (Figure 7B). The diversity of these additional accessory areas suggests that CMP conditions are not solely underpinned by a common neural nucleus but are also modulated by specific neural circuits that reflect the clinical heterogeneity of each condition. This finding reinforces the need to understand CMP as a multifaceted connectomic phenomenon, in which particular adaptations coexist and contribute to the persistence and complexity of the clinical profile.

The operating mechanism of the basic and additional accessory neural circuitries of CMP indicates a hybrid organization that integrates their components both in series and in parallel. This arrangement combines both primary (sensory and motor) and secondary activations because of pain (affective–emotional and cognitive–motivational) [4]. The serial functioning between the thalamus and somatosensory cortex in the basic neural circuitry supports somatotopic discrimination (i.e., anatomical location and intensity of pain). The somatosensory cortex and the motor cortex are activated in parallel [8, 9]; in addition, the thalamus projects to the insula and to the anterior cingulate cortex, which are areas of the SN [98]. This network is responsible for detecting and integrating stimuli that are biologically relevant to tissue integrity and survival, allowing chronic pain to be encoded not only as a sensation but as a high‐priority emotional experience [11, 98, 99]. Connectivity alterations within the SN have been identified as biomarkers in the transition between acute and chronic pain [99]. The cerebellum is activated in both serial chains with motor areas and in parallel interactions with the prefrontal cortex that contribute to cognitive modulation [38, 100]. Thus, the CMP basic neural circuitry figures as a dynamic and integrative connectome that coordinates multiple levels of processing (sensory–motor, affective–emotional, and cognitive–motivational) organized in a functional sequence that converts nociceptive signals into a conscious experience.

The additional accessory neural circuitries identified in some CMP subgroups present an organization predominantly parallel with the basic neural circuitry without replacing it. These parallel connections expand the CMP processing network and add dimensions related to reward, memory, descending pain modulation, and attention [99]. This integration between basic neural circuitry and additional accessory neural circuitries forms a dynamic connectome, whose parallel configuration underlies different clinical profiles of CMP and sustains individual variability in the perception of pain experience.

In summary, this dynamic connectomic model is characterized by intra‐axis serial chains integrated into a parallel organization between axes of pain experience processing. This hybrid arrangement ensures both serial nociceptive transmission and simultaneous co‐activation of multiple encephalon regions that maintain pain in different clinical conditions.

### 4.3. Recommendations for Further Studies

Although the present systematic review with ALE meta‐analysis has outlined, for the first time, the basic and additional accessory neural circuitries of CMP, the understanding of connectomic and neurobiological mechanisms related to CMP is still limited; thus, further studies on the following topics are encouraged: (1) Which thalamic nuclei/subnuclei actively participate in the basic neural circuitry? (2) How does the basic neural circuitry behave throughout CMP treatment? (3) Which are the morphological alterations in neural cells in these encephalon regions, and how do they occur? (4) Is there an imbalance between excitatory and inhibitory neuronal interconnections in the basic neural circuitry that justifies a greater excitability of the regions associated with pain perception? (5) Which cellular mechanisms are involved in the interconnectivity between basic and additional accessory neural circuitries? (6) How do the neural cells that make up neural circuits interconnect between cortical layers? These questions, which remain unanswered in the current literature, may be promising targets for future research.

### 4.4. | Limitations

The most notable limitation of this study was analyzing functional changes in the encephalon involving only 16 CMP conditions, whereas CMP conditions encompass approximately a total of 150 subgroups [101]. Nevertheless, we emphasize that this reflects the availability of current literature, which tends to address the most prevalent conditions. Therefore, although few CMP conditions were studied in this systematic review, when compared to the number of existing conditions, our study plays an important role because it provides a mapping of the encephalon structures that identifies the neural circuitries that underlies the clinical phenotype of individuals. The risk of bias in the studies eligible for this systematic review can also be considered a limitation, since, although primary studies contribute fundamental information about the neural circuitries of the CMP, methodological deficiencies highlight the need for greater control of the risk of bias in future research. Furthermore, our study also presents some limitations inherent to neuroimaging meta‐analyses, such as scanner configurations and sample characteristics, which can introduce variability in the results. At last, analyses of some CMP subgroups were also limited in our systematic review due to the small number of studies available for some CMP conditions (KOA, CAI, CWAD, AS, CINP, Hands OA, MIRCT, RA, and AKP).

#### 4.4.1. Deviations From the Protocol

Although the protocol was previously registered in the PROSPERO platform, minor methodological adaptations were made during the review process. Specifically, the analysis was restricted to fMRI studies employing sensorimotor or resting‐state stimuli, thereby increasing the homogeneity of the experimental paradigms. Additionally, the analysis of structural changes, originally planned in the protocol, was not performed to ensure greater consistency in the synthesis of data of FC changes.

## 5. Conclusions

Seven encephalon regions comprise the basic neural circuitry of CMP: (1) somatosensory cortex, (2) motor cortex, (3) anterior cingulate cortex, (4) insula, (5) prefrontal cortex, (6) thalamus, and (7) cerebellum, corresponding to an integrated connectomic hub that coordinates multiple levels of processing (sensory–motor, affective–emotional, and cognitive–motivational) organized in a functional sequence, culminating in the transformation of nociceptive signals into a conscious experience. Additionally, 13 other areas are specifically added to the basic neural circuitry in the distinct conditions of CMP, composing the additional accessory neural circuitries. These parallel connections expand the processing network and add dimensions related to reward, memory, descending pain modulation, and attention. This dynamic reorganization reflects a pattern of cross‐modal plasticity, in which an area of the encephalon originally dedicated to processing one type of sensory modality, or function, is recruited to process other sensory modalities or perform other functions. Such changes may represent maladaptive mechanisms, as they contribute to the persistence of pain or disability.

## Author Contributions

Jeffeson Hildo Medeiros de Queiroz contributed substantially to the conception and design of the study, PROSPERO registration, literature search strategy development, data acquisition, risk of bias assessment, preprocessing and standardization of neuroimaging coordinates, ALE meta‐analysis implementation, complementary statistical analyses, preparation of data analysis reports, figure design and refinement, drafting of the manuscript, and integration of all methodological and interpretative components. Marcio Gonçalves Corrêa, Gabriel Mesquita da Conceição Bahia, Rebeca da Costa Gomes, Thais Alves Lobão, Erica Miranda Sanches Aires, Evander de Jesus Oliveira Batista, Gláucia Mota Bragança, Marta Chagas Monteiro, and Carlomagno Pacheco Bahia assisted with data acquisition, risk of bias evaluation, and figure preparation. Carlomagno Pacheco Bahia contributed to the study conception and design, provided supervision, and critically revised the manuscript for important intellectual content.

## Funding

Carlomagno Pacheco Bahia was supported by the National Council for Scientific and Technological Development – CNPq (Grants 310054/2018‐4, 447835/2014‐9, 483404/2013‐6, 444967/2020‐6, 444982/2020‐5, and 407504/2025‐7) and the Brazilian Agency for Support and Evaluation of Graduate Education – CAPES (Grants PROCAD 21/2018). Marta Chagas Monteiro was supported by the National Institutes of Science, Technology and Innovation (PROBIAM Pharmaceuticals Amazonia – INCT/CNPq Grant 406819/2022‐0), the Brazilian Agency for Support and Evaluation of Graduate Education – CAPES (Grant 88882.461690/2019‐01), and the Fundação Amazônia Paraense de Amparo à Pesquisa (FAPESPA) (Grant 005/2016).

## Disclosure

All authors have reviewed and approved the final version of the manuscript. The funders had no role in study design, data collection and analysis, decision to publish, or preparation of the manuscript.

## Ethics Statement

This study is a systematic review with ALE meta‐analysis based on previously published data. No data were collected directly from humans or other animals.

## Conflicts of Interest

The authors declare no conflicts of interest.

## Supporting Information

Additional supporting information can be found online in the Supporting Information section.

## Supporting information


**Supporting Information 1** Contains the duly filled PRISMA 2020 checklist.

**Supporting Information 2** Presents the completed checklist for neuroimaging meta‐analyses, based on the recommendations proposed by Müller et al. [23].

**Supporting Information 3** Outlines the systematic review protocol, including PECO questions, hypothesis, eligibility criteria with their respective justifications, the PROSPERO register, and the full search strategies applied across all databases.

**Supporting Information 4** Table S1. (Risk of bias – New Castle–Ottawa) reports the methodological quality assessment of the included studies using the Newcastle–Ottawa Scale for cohort and case–control designs.

**Supporting Information 5** Table S2. (Data extraction and study characteristics) presents the extracted data from all included studies, including study design, sample characteristics, chronic musculoskeletal pain subtype, experimental paradigms, and individual results from each study selected.

**Supporting Information 6** Table S3. (Activated regions and frequency analysis) The additional accessory neural circuitries identified presents the activation of brain regions associated with chronic musculoskeletal pain conditions, as well as the frequency of activation in the identified areas.

**Supporting Information 7** Lists all peak activation coordinates extracted from the primary studies and used as input for the activation likelihood estimation meta‐analysis.

**Supporting Information 8** You will find the complete list of the 43 references corresponding to the studies selected in this systematic review.

**Supporting Information 9** Provides a comprehensive list of all abbreviations and acronyms used throughout the manuscript to facilitate clarity and readability.

## Data Availability

The data that support the findings of this study are available in the Supporting Information section of this article.
