# Supplementary material for: Connectomic Mapping of Chronic Musculoskeletal Pain: Neural Circuitries Identified Through a Systematic Review and ALE Meta‐Analysis
Source: Neural Plast. 2026 May 8;2026:5301861. doi: 10.1155/np/5301861 (PMC13155939; doi:10.1155/np/5301861)
Supplement: Supplementary file 2 — Supporting Information 2 Presents the completed checklist for neuroimaging meta‐analyses, based on the recommendations proposed by Müller et al. [23]. [file NP-2026-5301861-s003.docx]

**Checklist for neuroimaging meta-analyses**

The research question is specifically defined

YES, and it includes the following contrasts:

Each type of chronic musculoskeletal pain vs Controls

The literature search was systematic

YES, it included the following keywords in the following databases:

PubMed, Web of Science, and Scopus

Detailed inclusion and exclusion criteria are included

YES, and reasons of non-standard criterion were:

Dashboard (Supplementary material – Inclusion criteria)

Sample overlap was taken into account

YES, using the following method:

Overlapping samples were not considered (line 181). Sample overlap inflates the activation probability. Furthermore, sample overlap is not appropriate, as it violates the assumption of independence between experiments.

Turkeltaub PE, Eickhoff SB, Laird AR, Fox M, Wiener M, Fox P. Minimizing within-experiment and within-group effects in Activation Likelihood Estimation meta-analyses. Hum Brain Mapp. 2012 Jan;33(1):1-13.

All experiments use the same search coverage (state how brain coverage is assessed and how small volume corrections and conjunctions are taken into account)

YES, the search coverage is the following:

Rest-stating or sensorimotor stimuli

Studies are converted to a common reference space

YES, using the following conversion(s):

From line 170 to 181

Data extraction have been conducted by two investigators (ideal case) or double checked by the same investigator (state how double- checking was performed)

YES, the following authors:

JHMQ and GMCB checked inclusion criteria

JHMQ and CPB extracted coordinates

JHMQ and CPB extracted other info: authors, year of publication, CMP condition studied, study type (i.e., cohort or case-control), sample size in each group, risk of bias classification, outcomes, main results, and, when available in the selected studies, and the absolute frequency with which encephalon areas were activated in the different CMP conditions

JHMQ and GMCB double-checked the following data: risk of bias

The paper

table with at least the references, basic study description (e.g. for fMRI tasks, stimuli), contrasts and basic sample descriptions (e.g. size, mean age and gender distribution, specific characteristics) of the included studies, source of information (e.g. contact with authors), reference space

The study protocol was previously registered, and all analyses planned beforehand, including the methods and parameters used for inference, correction for multiple testing, etc

YES:

1. The meta-analysis was registered before starting the search at

PROSPERO (registration number CRD42022382309)

1. Any non-planned analyses are clearly stated as post-hoc or non-prespecified in the paper:

Lines 98 and 99

1. The meta-analysis used the default methods and parameters of the software, with the following exceptions:

From line 157 to 201

The meta- analysis includes diagnostics

YES, the following:

From line 157 to 201

YES, and also the following data:

Data collection – Supplementary material
