# Supplementary material for: Connectomic Mapping of Chronic Musculoskeletal Pain: Neural Circuitries Identified Through a Systematic Review and ALE Meta‐Analysis
Source: Neural Plast. 2026 May 8;2026:5301861. doi: 10.1155/np/5301861 (PMC13155939; doi:10.1155/np/5301861)
Supplement: Supplementary file 3 — Supporting Information 3 Outlines the systematic review protocol, including PECO questions, hypothesis, eligibility criteria with their respective justifications, the PROSPERO register, and the full search strategies applied across all databases. [file NP-2026-5301861-s007.docx]

**Connectomic Mapping of Chronic Musculoskeletal Pain: Neural Circuitries Identified Through a Systematic Review and ALE Meta-Analysis**

Jeffeson Hildo Medeiros de Queiroz | Gabriel Mesquita da Conceição Bahia | Marcio Gonçalves Corrêa | Rebeca da Costa Gomes | Thais Alves Lobão | Erica Miranda Sanches Aires | Evander de Jesus Oliveira Batista | Gláucia Mota Bragança | Marta Chagas Monteiro | Carlomagno Pacheco Bahia

**Federal University of Para - Belem – Pará, Brazil**

Postgraduate Program in Neuroscience and Cellular Biology

Laboratory of Neuroplasticity

1. **Purpose**

To characterize specific patterns of connectomic reorganization in different CMP subgroups.

1. **Hypothesis**

The hypothesis tested in this research proposes that each CMP condition exhibits distinct patterns of connectomic reorganization.

1. **PECO questions**

- 1 - Which encephalon regions (O) constitute the neural circuitry of humans (P) with CMP (E)?
- 2 - Which specific alterations are observed in this neural circuitry (O) in different CMP subgroups (E) in humans (P)?

1. **International Prospective Register of Systematic Reviews (PROSPERO) register:** CRD42022382309
2. **Search strategy on the databases**
   1. **PubMed**

(("Brain"[MeSH Terms] OR "Brain"[Title/Abstract] OR "Encephalon"[Title/Abstract] OR "central nervous systems"[Title/Abstract] OR "nervous system central"[Title/Abstract] OR "nervous systems central"[Title/Abstract] OR "systems central nervous"[Title/Abstract] OR "cerebrospinal axis"[Title/Abstract] OR ("Axi"[All Fields] AND "Cerebrospinal"[Title/Abstract]) OR "axis cerebrospinal"[Title/Abstract] OR ("Cerebrospinal"[All Fields] AND "Axi"[Title/Abstract])) AND ("musculoskeletal pain"[MeSH Terms] OR "musculoskeletal pain"[Title/Abstract] OR "musculoskeletal pains"[Title/Abstract] OR "pain musculoskeletal"[Title/Abstract] OR "pains musculoskeletal"[Title/Abstract] OR "chronic pain"[MeSH Terms] OR "chronic pain"[Title/Abstract] OR "chronic pains"[Title/Abstract] OR "pains chronic"[Title/Abstract] OR "pain chronic"[Title/Abstract] OR "widespread chronic pain"[Title/Abstract] OR "chronic pain widespread"[Title/Abstract] OR (("chronic pain"[MeSH Terms] OR ("Chronic"[All Fields] AND "Pain"[All Fields]) OR "chronic pain"[All Fields] OR ("Chronic"[All Fields] AND "Pains"[All Fields]) OR "chronic pains"[All Fields]) AND "Widespread"[Title/Abstract]) OR (("Pain"[MeSH Terms] OR "Pain"[All Fields]) AND "widespread chronic"[Title/Abstract]) OR (("Pain"[MeSH Terms] OR "Pain"[All Fields] OR "painful"[All Fields] OR "Pains"[All Fields] OR "pain s"[All Fields] OR "painfulness"[All Fields]) AND "widespread chronic"[Title/Abstract]) OR "widespread chronic pains"[Title/Abstract]) AND ("magnetic resonance imaging"[MeSH Terms] OR "magnetic resonance imaging"[Title/Abstract] OR "imaging magnetic resonance"[Title/Abstract] OR "nmr imaging"[Title/Abstract] OR "imaging nmr"[Title/Abstract] OR "tomography nmr"[Title/Abstract] OR "tomography mr"[Title/Abstract] OR "mr tomography"[Title/Abstract] OR "nmr tomography"[Title/Abstract] OR "steady state free precession mri"[Title/Abstract] OR "steady state free precession mri"[Title/Abstract] OR "Zeugmatography"[Title/Abstract] OR "imaging chemical shift"[Title/Abstract] OR "chemical shift imagings"[Title/Abstract] OR (("Image"[All Fields] OR "image s"[All Fields] OR "imaged"[All Fields] OR "imager"[All Fields] OR "imager s"[All Fields] OR "imagers"[All Fields] OR "Images"[All Fields] OR "Imaging"[All Fields] OR "imaging s"[All Fields] OR "Imagings"[All Fields]) AND "chemical shift"[Title/Abstract]) OR ((("magnetic resonance imaging"[MeSH Terms] OR ("Magnetic"[All Fields] AND "Resonance"[All Fields] AND "Imaging"[All Fields]) OR "magnetic resonance imaging"[All Fields] OR ("Shift"[All Fields] AND "Imaging"[All Fields] AND "Chemical"[All Fields]) OR "shift imaging chemical"[All Fields]) AND ("Shift"[All Fields] OR "shifted"[All Fields] OR "shifting"[All Fields] OR "shiftings"[All Fields] OR "shifts"[All Fields]) AND ("Image"[All Fields] OR "image s"[All Fields] OR "imaged"[All Fields] OR "imager"[All Fields] OR "imager s"[All Fields] OR "imagers"[All Fields] OR "Images"[All Fields] OR "Imaging"[All Fields] OR "imagings"[All Fields] OR "Imagings"[All Fields])) AND "Chemical"[Title/Abstract]) OR "chemical shift imaging"[Title/Abstract] OR "magnetic resonance image"[Title/Abstract] OR "image magnetic resonance"[Title/Abstract] OR "magnetic resonance images"[Title/Abstract] OR "resonance image magnetic"[Title/Abstract] OR "magnetization transfer contrast imaging"[Title/Abstract] OR "mri scans"[Title/Abstract] OR "mri scan"[Title/Abstract] OR "scan mri"[Title/Abstract] OR "scans mri"[Title/Abstract] OR (("tomographie"[All Fields] OR "Tomography"[MeSH Terms] OR "Tomography"[All Fields] OR "tomographies"[All Fields] OR "tomography s"[All Fields] OR "tomographys"[All Fields]) AND "proton spin"[Title/Abstract]) OR "proton spin tomography"[Title/Abstract] OR "fMRI"[Title/Abstract] OR (("magnetic resonance imaging"[MeSH Terms] OR ("Magnetic"[All Fields] AND "Resonance"[All Fields] AND "Imaging"[All Fields]) OR "magnetic resonance imaging"[All Fields] OR ("MRI"[All Fields] AND "Functional"[All Fields]) OR "mri functional"[All Fields]) AND "functional mri"[Title/Abstract]) OR "functional mris"[Title/Abstract] OR ("MRIs"[All Fields] AND "Functional"[Title/Abstract]) OR "functional magnetic resonance imaging"[Title/Abstract] OR "magnetic resonance imaging functional"[Title/Abstract] OR "spin echo imaging"[Title/Abstract] OR "echo imaging spin"[Title/Abstract] OR ((("Echo"[Journal] OR "Echo"[All Fields]) AND ("Image"[All Fields] OR "image s"[All Fields] OR "imaged"[All Fields] OR "imager"[All Fields] OR "imager s"[All Fields] OR "imagers"[All Fields] OR "Images"[All Fields] OR "Imaging"[All Fields] OR "imaging s"[All Fields] OR "Imagings"[All Fields])) AND "Spin"[Title/Abstract]) OR "imaging spin echo"[Title/Abstract] OR (("Image"[All Fields] OR "image s"[All Fields] OR "imaged"[All Fields] OR "imager"[All Fields] OR "imager s"[All Fields] OR "imagers"[All Fields] OR "Images"[All Fields] OR "Imaging"[All Fields] OR "imaging s"[All Fields] OR "Imagings"[All Fields]) AND "spin echo"[Title/Abstract]) OR ("Spin"[All Fields] AND "echo imagings"[Title/Abstract]))) AND (2002:2024[pdat])

**5.2 Web of Science**

(((((((((((((TS=(Musculoskeletal pain)) OR TS=(Chronic pain)) OR TS=(Musculoskeletal Pains)) OR TS=(Pain, Musculoskeletal)) OR TS=(Pains, Musculoskeletal)) OR TS=(Chronic Pains)) OR TS=(Pains, Chronic)) OR TS=(Pain, Chronic)) OR TS=(Widespread Chronic Pain)) OR TS=(Chronic Pain, Widespread)) OR TS=(Chronic Pains, Widespread)) OR TS=(Pain, Widespread Chronic)) OR TS=(Pains, Widespread Chronic)) OR TS=(Widespread Chronic Pains) AND (((((((((TS=(Brain)) OR TS=(Encephalon)) OR TS=(Central Nervous Systems)) OR TS=(Nervous System, Central)) OR TS=(Nervous Systems, Central)) OR TS=(Systems, Central Nervous)) OR TS=(Cerebrospinal Axis)) OR TS=(Axi, Cerebrospinal)) OR TS=(Axis, Cerebrospinal)) OR TS=(Cerebrospinal Axi) AND ((((((((((((((((((((((((((((((((((((((((TS=(Magnetic Resonance Imaging)) OR TS=(Imaging, Magnetic Resonance)) OR TS=(NMR Imaging)) OR TS=(Imaging, NMR)) OR TS=(Tomography, NMR)) OR TS=(Tomography, MR)) OR TS=(MR Tomography)) OR TS=(NMR Tomography)) OR TS=(Steady-State Free Precession MRI)) OR TS=(Steady State Free Precession MRI)) OR TS=(Zeugmatography)) OR TS=(Imaging, Chemical Shift)) OR TS=(Chemical Shift Imagings)) OR TS=(Imagings, Chemical Shift)) OR TS=(Shift Imaging, Chemical)) OR TS=(Shift Imagings, Chemical)) OR TS=(Chemical Shift Imaging)) OR TS=(Magnetic Resonance Image)) OR TS=(Image, Magnetic Resonance)) OR TS=(Magnetic Resonance Images)) OR TS=(Resonance Image, Magnetic)) OR TS=(Magnetization Transfer Contrast Imaging)) OR TS=(MRI Scans)) OR TS=(MRI Scan)) OR TS=(Scan, MRI)) OR TS=(Scans, MRI)) OR TS=(Tomography, Proton Spin)) OR TS=(Proton Spin Tomography)) OR TS=(fMRI)) OR TS=(MRI, Functional)) OR TS=(Functional MRI)) OR TS=(Functional MRIs)) OR TS=(MRIs, Functional)) OR TS=(Functional Magnetic Resonance Imaging)) OR TS=(Magnetic Resonance Imaging, Functional)) OR TS=(Spin Echo Imaging)) OR TS=(Echo Imaging, Spin)) OR TS=(Echo Imagings, Spin)) OR TS=(Imaging, Spin Echo)) OR TS=(Imagings, Spin Echo)) OR TS=(Spin Echo Imagings)

**5.3 Scopus**

TITLE-ABS-KEY ( "magnetic resonance imaging" ) OR TITLE-ABS-KEY ( "imaging, magnetic resonance" ) OR TITLE-ABS-KEY ( "nmr imaging" ) OR TITLE-ABS-KEY ( "imaging, nmr" ) OR TITLE-ABS-KEY ( "tomography, nmr" ) OR TITLE-ABS-KEY ( "tomography, mr" ) OR ( "mr tomography" ) OR ( "nmr tomography" ) OR TITLE-ABS-KEY ( "steady-state free precession mri" ) OR TITLE-ABS-KEY ( "steady state free precession mri" ) OR TITLE-ABS-KEY ( "zeugmatography" ) OR TITLE-ABS-KEY ( " imaging, chemical shift" ) OR TITLE-ABS-KEY ( "chemical shift imagings" ) OR TITLE-ABS-KEY ( "imagings, chemical shift" ) OR TITLE-ABS-KEY ( "shift imaging, chemical" ) OR TITLE-ABS-KEY ( "shift imagings, chemical" ) OR TITLE-ABS-KEY ( "chemical shift imaging" ) OR TITLE-ABS-KEY ( "Magnetic Resonance Image" ) OR TITLE-ABS-KEY ( "image, magnetic resonance" ) OR TITLE-ABS-KEY ( "magnetic resonance images" ) OR TITLE-ABS-KEY ( "Resonance Image, Magnetic" ) OR TITLE-ABS-KEY ( "magnetization transfer contrast imaging" ) OR TITLE-ABS-KEY ( "MRI Scans" ) OR TITLE-ABS-KEY ( "MRI Scan" ) OR TITLE-ABS-KEY ( "Scan, MRI " ) OR TITLE-ABS-KEY ( "scans, mri" ) OR TITLE-ABS-KEY ( "tomography, proton spin" ) OR TITLE-ABS-KEY ( "proton spin tomography" ) OR TITLE-ABS-KEY ( "fmri" ) OR TITLE-ABS-KEY ( "mri, functional" ) OR TITLE-ABS-KEY ( "functional mri" ) OR TITLE-ABS-KEY ( "functional mris" ) OR TITLE-ABS-KEY ( "mris, functional" ) OR TITLE-ABS-KEY ( "functional magnetic resonance imaging" ) OR TITLE-ABS-KEY ( "magnetic resonance imaging, functional" ) OR TITLE-ABS-KEY ( "spin echo imaging" ) OR TITLE-ABS-KEY ( "echo imaging, spin" ) OR TITLE-ABS-KEY ( "echo imagings, spin" ) OR TITLE-ABS-KEY ( "Imaging, spin echo" ) OR TITLE-ABS-KEY ( "imagings, spin echo" ) OR TITLE-ABS-KEY ( "spin echo imagings" ) AND TITLE-ABS-KEY ( "brain" ) OR TITLE-ABS-KEY ( "encephalon" ) OR TITLE-ABS-KEY ( "central nervous system" ) OR TITLE-ABS-KEY ( "central nervous systems" ) OR TITLE-ABS-KEY ( "nervous system, central" ) OR TITLE-ABS-KEY ( "nervous systems, central" ) OR ( "systems, central nervous" ) OR ( "cerebrospinal axis" ) OR TITLE-ABS-KEY ( "axi, cerebrospinal" ) OR TITLE-ABS-KEY ( "axis, cerebrospinal" ) OR TITLE-ABS-KEY ( "cerebrospinal axi" ) AND TITLE-ABS-KEY ( "musculoskeletal pain" ) OR TITLE-ABS-KEY ( "musculoskeletal pains" ) OR TITLE-ABS-KEY ( "pain, musculoskeletal" ) OR TITLE-ABS-KEY ( "pains, musculoskeletal" ) OR TITLE-ABS-KEY ( "chronic pain" ) OR TITLE-ABS-KEY ( "chronic pains" ) OR TITLE-ABS-KEY ( "pains, chronic" ) OR TITLE-ABS-KEY ( "pain, chronic" ) OR TITLE-ABS-KEY ( "widespread chronic pain" ) OR TITLE-ABS-KEY ( "chronic pain, widespread" ) OR TITLE-ABS-KEY ( "chronic pains, widespread" ) OR TITLE-ABS-KEY ( "pain, widespread chronic" ) OR TITLE-ABS-KEY ( "pains, widespread chronic" ) OR ( "widespread chronic pains" ) AND PUBYEAR > 2001 AND PUBYEAR < 2024

1. **Inclusion criteria and their respective justifications**

- Cohort studies or case-control studies

These study designs allow for structured comparisons between patients and controls, better control of confounding factors, and greater methodological rigor. They are also the most consistent study types in the functional magnetic resonance imaging literature for coordinate-based analyses, reducing heterogeneity.

- Performed in adult human participants

Including only adults removes variations associated with neurodevelopment and ensures greater stability of brain architecture and connectivity. This increases the comparability of findings and the validity of inferences between groups.

- Using functional magnetic resonance imaging

Functional magnetic resonance imaging offers high spatial resolution and standardized methods for measuring brain activity and connectivity, essential for identifying functional changes in chronic pain. It also provides coordinates compatible with ALE meta-analyses.

- Resting state or with sensorimotor stimulations

Resting-state studies demonstrate intrinsic networks of functional connectivity in chronic pain, while sensorimotor-nociceptive paradigms capture evoked encephalon processing and motor aspects relevant to musculoskeletal pain. Both broaden the understanding of the circuits involved.

- Published between 2002 and 2025

Since 2002, modern functional magnetic resonance imaging protocols, normalization for MNI/Talairach, and reproducible voxel-wise statistics have been consolidated. Limiting the range time ensures methodological quality and compatibility with coordinate-based meta-analyses.

References: Friston, K. J. (2003). Statistical parametric mapping. In R. S. J. Frackowiak, K. J. Friston, C. Frith, R. Dolan, & J. C. Mazziotta (Eds.), *Human brain function* (2nd ed., pp. 599–634). Academic Press. <https://doi.org/10.1016/B978-012264841-0/50033-0>

Brett, M., Johnsrude, I. S., & Owen, A. M. (2002). The problem of functional localization in the human brain. *Nature Reviews Neuroscience, 3*(3), 243–249. <https://doi.org/10.1038/nrn756>).

- English language

The highest quality functional magnetic resonance imaging literature is predominantly published in English, ensuring full access to data and editorial standardization. This reduces interpretation biases and ensures accuracy in the extraction and analysis of coordinates.

- Report brain functional alterations in patients with chronic musculoskeletal pain (duration ≥ 3 months), excluding studies with comorbidities that could confound brain alterations (e.g., neurological or major psychiatric disorders)

The criterion of ≥3 months follows the International Association for Study of Pain definition for chronic pain, and excluding comorbidities avoids confounding brain changes. Thus, the findings reflect functional changes specific to chronic musculoskeletal pain, and not to other disorders.

- Specifying the type of chronic MSP for each exposed group

Chronic musculoskeletal pain encompasses heterogeneous conditions; specifying the type allows for stratified analyses, reduces heterogeneity, and improves accuracy in identifying underlying neural circuitry. This strengthens connectomic interpretation.

1. **Risk of bias:** Newcastle-Ottawa scale for cohort and case-control studies
2. **Search strategy results and screening**

| **PubMed** | 1,237 |
| --- | --- |
| **Web of Science** | 1,906 |
| **Scopus** | 2,300 |
| **Total in the databases** | 5,443 |
| Duplicates deleted before screening | 1,954 |
| Papers for screening | 3,489 |
| **Excluded by title and abstract** | 3.189 |
| Wrong population | 1,173 |
| Wrong study design | 948 |
| Wrong outcome | 300 |
| Wrong publication type | 252 |
| Case reports | 200 |
| Not humans | 116 |
| Not English language | 58 |
| Publication date (≤ 2001) | 46 |
| Pilot studies | 40 |
| Editorials | 22 |
| Protocols | 19 |
| Commentaries | 6 |
| It does not determine a pathology | 8 |
| Preface | 1 |
| Included by title and abstract | 300 |
| **Excluded by full text** | 257 |
| Wrong outcome | 118 |
| Wrong study design | 66 |
| Wrong population | 23 |
| It does not determine a type of pathology | 27 |
| Wrong experimental paradigm | 14 |
| Confounding variables (e.g., major psychiatric illness or neurological illness) | 4 |
| Preliminary studies | 4 |
| Pilot study | 1 |
| **Selected** | 43 |
| Cohorts | 4 |
| Case-controls | 39 |
| For the meta-analysis | 36 |

1. **Ethics Statement**

# This study is a systematic review with ALE meta-analysis based on previously published data. No data were collected directly from humans or other animals.

# **Competing interests**

The authors declare that they have no commercial or financial relationships that could be perceived as potential conflicts of interest related to this study.

# **Author Contributions**

JHMQ contributed substantially to the conception and design of the study, PROSPERO registration, literature search strategy development, data acquisition, risk of bias assessment, preprocessing and standardization of neuroimaging coordinates, ALE meta-analysis implementation, complementary statistical analyses, preparation of data analysis reports, figure design and refinement, drafting of the manuscript, and integration of all methodological and interpretative components. MGC, GMCB, RCG, TAL, EMSA, EJOB, GMB, MCM, and CPB assisted with data acquisition, risk of bias evaluation, and figure preparation. CPB contributed to the study conception and design, provided supervision, and critically revised the manuscript for important intellectual content. All authors reviewed and approved the final version of the manuscript.

1. **Funding**

CPB: National Council for Scientific and Technological Development – CNPq (grants no. 310054/2018-4, 447835/2014- 9, 483404/2013-6, 444967/2020-6, 444982/2020-5, and 407504/2025-7), and the Brazilian Agency for Support and Evaluation of Graduate Education – CAPES (grants PROCAD 21/2018). MCM: National Institutes of Science, Technology and Innovation (PROBIAM Pharmaceuticals Amazonia - INCT/CNPq grant 406819/2022- 0), the Brazilian Agency for Support and Evaluation of Graduate Education – CAPES (88882.461690/2019-01), and the Fundação Amazônia Paraense de Amparo à Pesquisa (FAPESPA) grant 005/2016. The funders had no role in study design, data collection and analysis, decision to publish, or preparation of the manuscript.
