# Supplementary material for: Connectomic Mapping of Chronic Musculoskeletal Pain: Neural Circuitries Identified Through a Systematic Review and ALE Meta‐Analysis
Source: Neural Plast. 2026 May 8;2026:5301861. doi: 10.1155/np/5301861 (PMC13155939; doi:10.1155/np/5301861)
Supplement: Supplementary file 7 — Supporting Information 7 Lists all peak activation coordinates extracted from the primary studies and used as input for the activation likelihood estimation meta‐analysis. [file NP-2026-5301861-s001.docx]

**Connectomic Mapping of Chronic Musculoskeletal Pain: Neural Circuitries Identified Through a Systematic Review and ALE Meta-Analysis**

Jeffeson Hildo Medeiros de Queiroz | Gabriel Mesquita da Conceição Bahia | Marcio Gonçalves Corrêa | Rebeca da Costa Gomes | Thais Alves Lobão | Erica Miranda Sanches Aires | Evander de Jesus Oliveira Batista | Gláucia Mota Bragança | Marta Chagas Monteiro | Carlomagno Pacheco Bahia

**X, Y, and Z coordinates from primary articles**

**# 1 - Low back pain**

// Referece=MNI152

// Tu et al., 2019: cLBP. vs. Controls

// Subjects=60

-5 50 2

-12 -65 36

-5 -54 51

6 -53 53

-7 41 -14

-48 -58 44

-5 -59 29

-6 -23 33

-5 47 44

-54 -7 32

54 -29 39

37 -26 54

-43 -20 57

-5 -2 69

-5 -26 68

-19 -8 56

18 -52 65

-28 11 -1

6 5 12

43 6 -11

-5 32 29

-16 54 21

-5 17 62

-43 -11 8

43 14 29

-34 57 6

30 42 33

-42 -4 44

45 39 14

// Reference=MNI152

// Shen et al., 2019: cLBP. vs. Controls

// Subjects=164

46 -30 50

24 -12 54

-48 -58 42

6 20 30

6 4 60

54 10 12

-60 8 -2

-48 -58 46

54 10 12

-2 -62 60

// Reference=MNI152

// Giesecke et al., 2004: cLBP. vs. Controls

// Subjects=38

58 -16 46

57 -23 16

-68 -14 10

45 -49 62

-34 -56 -29

58 -22 45

64 -15 11

50 -51 64

50 -25 19

-66 -17 10

-35 -61 20

// Reference=MNI152

// Ma et al., 2020: cLBP. vs. Controls

// Subjects=46

-21 -75 -42

6 -69 -15

24 -18 -27

-33 -54 -12

-18 24 9

9 -63 18

-48 6 54

-33 -54 63

-45 -27 66

-32 25 21

-32 25 9

// Reference=MNI152

// Hotz-Boendermaker et al., 2016: cLBP. vs. Controls

// Subjects=26

18.9 -34 65.6

18.9 -34.0 65.4

18.6 -34.2 65.4

-43.0 -32.1 12.1

-42.2 -31.8 12.3

-42.2 -26.9 12.4

47.7 -26.9 13.9

47.8 -26.9 14.2

47.4 -26.8 13.9

20 -34 68

46 -12 -16

-48 -32 8

// Reference=MNI152

// Baliki et al., 2014: cLBP. vs. Controls

// Subjects=87

-38 10 -12

2 36 22

-4 58 2

2 -56 26

// Reference=MNI152

// Zhu et al., 2024: cLBP. vs. Controls

// Subjects=112

51 36 -1

48 35 13

15 -22 71

-53 -31 23

5 -21 61

-13 -20 73

36 18 1

-56 -14 16

26 -23 -27

61 -40 -17

9 20 -19

23 36 -18

-6 -5 58

-16 -24 6

-18 -23 4

29 -27 -10

39 -7 8

39 -2 -9

-21 -35 68

56 -10 15

12 -14 1

22 -12 20

16 -85 34

-51 -33 42

31 -54 53

-21 -35 68

60 -53 3

-32 14 -34

54 4 9

-26 60 -6

10 -14 14

3 -13 5

22 8 -1

15 8 -9

8 -90 12

7 -76 11

10 -85 -9

39 -7 8

53 -54 25

47 -35 45

// Reference=MNI152

// Zhang et al., 2024: cLBP. vs. Controls

// Subjects=38

-18 51 -9

-27 21 12

-45 -51 -18

-39 -21 21

-36 -30 -18

-12 -63 21

-36 0 -18

-9 -105 -12

-39 -42 6

-15 -12 30

-9 -57 -21

36 18 0

51 -6 51

9 12 57

42 -3 -21

63 -39 18

15 -45 12

9 18 36

51 -27 24

21 -66 -51

-42 -51 18

-57 -33 1

// Reference=MNI152

// Mao et al., 2023: cLBP. vs. Controls

// Subjects=104

-14 10 58

14 4 6

-38 14 -12

42 18 -10

8 -20 -30

94 133 129

129 139 61

107 129 79

100 107 43

// Reference=MNI152

// Fan et al., 2023: cLBP. vs. Controls

// Subjects=112

0 15 36

0 -78 39

-39 15 0

-21 -39 75

51 15 33

3 -84 18

-9 -3 -15

// Reference=MNI152

// Chen et al., 2023: cLBP. vs. Controls

// Subjects=46

-24 -69 -33

26 -71 -33

-9 -27 60

9 1 58

// Reference=MNI152

// Hu et al., 2024: cLBP. vs. Controls

// Subjects=83

-12 51 -24

12 33 -21

-39 0 -9

**# 2 - Fibromyalgia**

// Reference=MNI152

// Park et al., 2022: FM. vs. Controls.

// Subjects=69

4 50 -4.5

-4 50 -4.5

10 12 -8

2 52 -2

-30 -20 -18

24 -2 -16

-8 14 2

8 20 2

-16 4 -10

-32 20 -4

36 20 -6

-6 -16 8

4 -14 8

-10 8 -4

2 44 20

0 44 10

// Reference=MNI152

// Schreiber et al., 2017: FM. vs. Controls.

// Subjects=70

20 2 -24

20 -2 -22

38 18 -42

20 -2 -34

26 -10 -22

40 0 -42

54 -56 -20

-16 62 20

48 -74 -32

8 36 -8

-2 2 72

-22 40 50

20 64 24

-4 -6 42

-28 -8 66

-52 -6 50

-36 -22 -22

-46 -72 -24

-28 12 -16

-6 44 8

16 44 50

-32 -20 42

20 -14 -26

-10 28 28

22 -20 -16

-14 -8 -20

30 -24 -24

-16 -12 -18

8 8 -10

-6 14 -4

-18 -6 -32

16 -6 -20

0 -60 62

8 -66 66

-24 16 -8

-56 36 0

-20 -4 -16

-12 20 2

-22 -16 -20

-36 -10 6

14 -98 14

-9 -94 4

-4 52 24

0 48 14

// Reference=MNI152

// Gieseck et al., 2004: FM. vs. Controls.

// Subjects=38

60 -18 46

65 -29 14

-62 -16 8

38 -51 66

-32 -55 -30

58 -16 46

68 -23 16

41 -44 52

42 5 6

2 16 43

-71 -16 8

-36 -60 -31

// Reference=MNI152

// Napadow et al., 2010: FM. vs. Controls

// Subjects=36

-34 6 10

-36 -8 18

-46 -30 22

-52 -34 28

30 -44 44

42 14 8

38 -10 6

60 26 -12

28 26 -6

-36 -8 10

-32 -20 14

18 -20 -18

24 16 -6

-2 -26 -10

12 -26 -16

-2 -30 -28

// Reference=MNI152

// Tong et al., 2023: FM. vs. Controls.

// Subjects=66

48 -26 46

48 -28 48

1. -26 48

**# 3 - Low back pain related leg pain**

// Reference=MNI152

// Wei et al., 2022: LBPRLP. vs. Controls.

// Subjects=70

-36 6 27

// Reference=MNI152

// Zhou et al., 2019: LBPRLP. vs. Controls.

// Subjects=51

6 -36 -60

-6 -12 -36

57 -63 -18

12 12 -12

-21 57 9

-6 30 15

0 51 33

21 -30 -33

-21 57 9

0 -48 33

12 15 -15

-30 45 9

-6 -12 -36

12 12 -12

-21 45 18

24 45 18

24 57 -3

-6 27 15

0 -66 60

3 -33 -57

0 12 -21

57 -63 -18

-12 12 -9

-18 54 9

-51 -51 36

-3 -51 36

9 -12 -33

-15 63 -6

51 -48 21

-3 -48 33

15 -81 51

-54 -51 12

0 12 -21

18 -81 -48

-30 27 57

15 15 -18

-9 0 72

0 27 -27

3 12 -24

-21 57 12

-51 -51 36

0 -45 45

21 -30 -33

-24 60 12

-39 -57 45

-18 -39 -51

-36 30 45

3 -36 -60

9 -15 -33

12 14 -12

54 -51 21

-30 48 12

9 -90 6

0 -48 33

15 -42 -42

-15 21 15

51 -12 15

21 21 6

0 -63 21

-57 -51 12

51 -57 21

// Reference=MNI152

// Pei et al., 2020: LBPRLP. vs. Controls.

// Subjects=60

30 27 36

30 24 39

36 48 27

33 42 18

60 -39 27

-24 -48 57

1. -18 36

**# 4 - Temporomandibular disorder**

// Reference=MNI152

// He et al., 2018: TMD. vs. Controls.

// Subjects=50

24 12 3

-24 3 12

-39 15 -9

-6 33 21

6 39 9

-21 9 0

-6 33 21

24 -18 63

-54 -33 -15

-54 -33 18

-48 0 54

-12 12 9

9 -15 9

-6 -12 6

-57 -33 0

-12 12 9

24 15 6

-24 3 15

-39 15 -9

6 39 9

-6 33 21

-45 21 -12

-33 3 -3

-9 30 24

36 9 0

-9 33 21

-39 30 -6

18 -21 57

-6 30 51

-48 0 51

-42 0 54

-12 42 24

-12 12 9

9 -15 9

-6 -12 6

-57 -33 0

-12 15 -3

-54 21 9

-36 45 9

// Reference=MNI152

// Chen et al., 2022: TMD vs. Controls

// Subjects=51

12 -39 0

-3 -48 0

3 -42 33

18 24 66

-18 -12 78

24 -18 78

30 -87 -33

31.5 -43 6

-1 -49 -4

// Reference=MNI152

// Nebel et al., 2010: TMD vs. Controls

// Subjects=25

-52 -36 56

-54 -18 54

-52 -20 14

-48 -22 12

50 -20 16

46 -20 10

-34 14 4

-40 12 -10

-40 -10 -6

-24 -10 -12

36 18 2

32 26 2

-12 4 -4

6 -18 -16

4 -18 0

-16 -22 2

56 -32 18

-40 -54 44

58 -46 50

-4 14 44

8 16 46

4 38 14

-6 32 18

38 46 2

48 34 26

-44 -2 12

-48 -4 8

-60 -22 24

-60 -26 46

-52 -30 56

-8 -28 -2

12 -24 8

-54 -22 52

-46 -18 52

-54 -30 14

-50 -22 14

-44 -34 20

-44 -24 8

44 -22 6

48 -24 18

-48 -10 -8

-6 2 40

4 8 40

-24 -10 -12

**# 5 - Chronic myofascial pain**

// Reference=MNI152

// Song et al., 2021: MP. vs. Controls.

// Subjects=46

-54 6 -18

55 6 -29

27 6 -27

22 -11 -11

-1 5 5

16 44 41

7 -33 -3

-9 -72 -3

6 -75 -3

3 -50 19

-40 -63 41

54 -57 33

-9 -72 -3

6 -75 -3

3 -50 19

-40 -63 41

54 -57 33

-54 3 -10

-60 -12 -20

44 45 17

0 56 15

2 -32 -7

66 -19 -19

49 -48 -20

22 -64 20

-23 -54 -10

21 60 -9

-48 6 2

-66 -39 39

-24 -57 -6

33 0 -27

26 -1 -29

-56 -36 -11

9 -34 -2

-21 15 56

// Reference=MNI152

// Niddam et al., 2008: MP. vs. Controls.

// Subjects=32

-48 19 8

-53 -12 27

-52 -27 47

-60 -11 18

-52 -12 25

-54 -28 48

-60 -11 18

-30 -39 2

-23 -36 -13

-15 -25 8

-38 -19 -7

-21 -9 1

-10 -72 38

-23 -36 -13

-19 -16 2

-40 -14 -3

51 16 3

55 -23 23

54 -24 35

50 -38 41

47 -70 23

40 16 -17

44 4 16

38 -4 9

59 -14 27

52 -25 34

42 -2 15

33 -62 41

33 -30 -9

16 -78 38

35 -60 36

33 -28 -8

1. -15 3

**# 6 - Chronic cervical spondylotic pain**

// Reference=MNI152

// Yang et al.,2020: CCSP. vs. Controls.

// Subjects=61

5 -33 48

53 -27 8

6 -71 51

15 39 -12

6 54 -24

// Reference=MNI152

// Zhang, Wang, and Guo 2024: CCSP. vs. Controls.

// Subjects=60

-3 12 30

-45 9 -6

-60 3 6

1. -36 48

**# 7 - Adhesive capsulitis**

// Reference=MNI152

// Li et al., 2023: AC. vs. Controls.

// Subjects=106

-3 -6 9

-3 -12 0

3 -15 12

1 12 30

-54 -24 54

// Reference=MNI152

// Li et al., 2024: AC. vs. Controls.

// Subjects=104

-18 -3 -12

3 63 0

6 48 1

1. 54 15

**# 8 - Knee Osteoarthritis**

**//** Reference=MNI152

// Baliki et al., 2014: KOA. vs. Controls

// Subjects=54

-4 58 2

2 36 22

42 14 -6

-38 10 -12

-56 -36 26

**# 9 - Chronic Ankle Instability**

// Reference=MNI152

// Shen et al., 2022: CAI. vs. Controls

// Subjects=64

48 -24 60

42 51 -6

42 -9 66

39 27 54

0 45 9

60 -18 48

-60 -18 -9

-54 -51 36

**#10 - Ankylosing spondylitis**

// Rerefence=MNI152152

// Hemington1 et al., 2015: AS vs. Controls

// Subjects=40

-40 12 -2

54 -30 44

-2 -66 12

2 6 24

-8 -32 46

6 -32 52

6 -66 60

6 -32 -38

-2 -68 20

**# 11 - Hands osteoarthritis**

// Reference=MNI152152

// Sofat et al., 2013: Hands OA. vs. Controls.

// Subjects=13

-1 -4 52

-46 1 24

-44 -24 52

-38 -24 54

-32 -26 48

2 -4 60

58 2 58

59 9 -6

52 4 3

49 0 18

46 10 0

43 -3 6

-40 -23 55

-40 -21 60

-40 -14 46

-39 -18 53

-39 -16 53

-3 -9 56

**# 12 - Rotator cuff tear**

// Reference=MNI152

// Conboy et al., 2021: RCT. vs. controls

// Subjects=21

-48 -31 50

-56 -67 32

48 -36 10

-23 -28 5

36 -36 -29

56 -53 33

-46 -30 48

52 -38 4

-3 -19 67

55 -65 2

36 -57 -2

1. -86 9

**# 13 - Anterior knee pain**

// Reference=MNI152

// Li et al., 2023: AKP. vs. Controls.

// Subjects=106

-3 -6 9

-3 -12 0

3 -15 12

1 12 30

-54 -24 54

// Reference=MNI152

// Li et al., 2024: AKP. vs. Controls.

// Subjects=104

-18 -3 -12

3 63 0

6 48 1

1. 54 15

**# 14 - Anterior Knee Pain with Catastrophizing**

// Reference=MNI152

// Sanchis-Alfonso et al., 2023: AKP_C. vs. Controls.

// Subjects=45

22 6 -2

22 -88 -38

18 -40 -54

41 38 30

15 -24 15

-33 -59 -21
