# Supplementary material for: Connectomic Mapping of Chronic Musculoskeletal Pain: Neural Circuitries Identified Through a Systematic Review and ALE Meta‐Analysis
Source: Neural Plast. 2026 May 8;2026:5301861. doi: 10.1155/np/5301861 (PMC13155939; doi:10.1155/np/5301861)
Supplement: Supplementary file 8 — Supporting Information 8 You will find the complete list of the 43 references corresponding to the studies selected in this systematic review. [file NP-2026-5301861-s002.docx]

# **Participant Inclusivity**

# This study is a systematic review with ALE meta-analysis based on previously published data. No data were collected directly from humans or other animals. Therefore, I confirm that this stipulation is not applicable, as my article reports no animal’s subject research.
