# Supplementary material for: Connectomic Mapping of Chronic Musculoskeletal Pain: Neural Circuitries Identified Through a Systematic Review and ALE Meta‐Analysis
Source: Neural Plast. 2026 May 8;2026:5301861. doi: 10.1155/np/5301861 (PMC13155939; doi:10.1155/np/5301861)
Supplement: Supplementary file 9 — Supporting Information 9 Provides a comprehensive list of all abbreviations and acronyms used throughout the manuscript to facilitate clarity and readability. [file NP-2026-5301861-s004.docx]

**Connectomic Mapping of Chronic Musculoskeletal Pain: Neural Circuitries Identified Through a Systematic Review and ALE Meta-Analysis**

Jeffeson Hildo Medeiros de Queiroz | Gabriel Mesquita da Conceição Bahia | Marcio Gonçalves Corrêa | Rebeca da Costa Gomes | Thais Alves Lobão | Erica Miranda Sanches Aires | Evander de Jesus Oliveira Batista | Gláucia Mota Bragança | Marta Chagas Monteiro | Carlomagno Pacheco Bahia

**Abbreviations list**

| **Abbreviations** | **Full term** |
| --- | --- |
| AC | Adhesive Capsulitis |
| AKP | Anterior Knee Pain |
| ALE | Activation Likelihood Estimation |
| ALFF | Amplitude of Low-Frequency Fluctuation |
| AS | Ankylosing Spondylitis |
| BOLD | Blood-Oxygen-Level-Dependent signal |
| CAI | Chronic Ankle Instability |
| CAPES | Coordenação de Aperfeiçoamento de Pessoal de Nível Superior |
| CCSP | Chronic Cervical Spondilotic Pain |
| CINP | Chronic Idiopathic Neck Pain |
| CMP | Chronic Musculoskeletal Pain |
| CLBP | Chronic Low Back Pain |
| CWAD | Chronic Whiplash-Associated Disorders |
| DMN | Default Mode Network |
| FC | Functional Connectivity |
| FM | Fibromyalgia |
| fMRI | Functional Magnetic Resonance Imaging |
| FWE | Family-Wise Error |
| GBD | Global Burden of Diseases |
| Hand OA | Hand Osteoarthritis |
| ICD | International Classification of Diseases |
| KOA | Knee Osteoarthritis |
| LBPRLP | Low back Pain Related Leg Pain |
| M1 | Primary Motor Cortex |
| M2 | Secondary Motor Cortex |
| MeSH | Medical Subject Headings |
| MIRCT | Massive Irreparable Rotator Cuff Tear |
| MNI | Montreal Neurological Institute |
| MP | Myofascial Pain |
| NOS | Newcastle–Ottawa Scale |
| OSF | Open Science Framework |
| PAG | Periaqueductal Gray Matter |
| PECO | Population/Problem, Exposure, Control/Comparison, and Outcomes |
| PFC | Prefrontal Cortex |
| PROSPERO | International Prospective Register of Systematic Reviews |
| PRISMA | Preferred Reporting Items for Systematic Reviews and Meta-Analyses |
| RA | Rheumatoid Arthritis |
| S1 | Primary Somatosensory Cortex |
| S2 | Secondary Somatosensory Cortex |
| SN | Salience Network |
| TMD | Temporomandibular Disorder |
